# Multidisciplinary study of the health and nutritional status of persons living in households at risk of poverty with children in Germany (MEGA_kids): Study design and methods

**DOI:** 10.1177/02601060241242159

**Published:** 2024-03-27

**Authors:** Anja Simmet, Janine Ehret, Romy Schleicher, Michael Teut, Gerrit Hummel, Andreas Bschaden, Nanette Stroebele-Benschop

**Affiliations:** 1 Department of Nutritional Psychology, Institute of Nutritional Medicine, 26558 University of Hohenheim, Stuttgart, Germany; 2 Institute of Social Medicine, Epidemiology and Health Economics, 14903 Charité – Universitätsmedizin Berlin, Berlin, Germany

**Keywords:** Food insecurity, poverty, nutrition, children, protocol, families

## Abstract

**Background:** In Germany, the nutritional situation of adults and children living in households at risk of poverty has been insufficiently studied so far. **Aim:** The aim of the mixed-methods study MEGA_kids is to gain a deeper understanding of the nutritional situation including socioeconomic, behavioral, and attitudinal factors and health characteristics among persons living in families at risk of poverty. **Method:** MEGA_kids is a mixed-methods cross-sectional study consisting of four modules combining quantitative and qualitative methods. The first module (A) applies self-administered questionnaires to assess the individual's diet, household food insecurity, and several other factors among adults and children of 500 households. Cash receipts are used to assess household's food expenses. For the second module (B), a semistructured interview guide is used to identify factors influencing food security and nutritional quality from the perspective of a subsample of module A (n = 20). The third module (C) applies the participatory World Café technique to explore experiences and generate ideas for tailored support measures for a healthy diet from the perspective of 40 parents participating in module A. Finally, the fourth module (D) investigates the knowledge and usage of existing nutrition-related preventive measures among 200 parents at risk of poverty by using an online questionnaire. **Conclusion:** By providing a comprehensive picture of nutritional aspects of families living at risk of poverty, MEGA_kids will guide officials to target and prioritize public health nutrition measures, inform policy makers to implement and improve healthy policies and, finally, identify research gaps to be prioritized.

## Introduction

In 2021, nearly 16% of the population were at risk of poverty in Germany, that is, roughly 13 million people have an equivalized disposable income below the at risk of poverty threshold, which is set at 60% of the national median equivalized disposable income after social transfers. People at a particular high risk of poverty include children, single parents, households with three or more children and people aged at least 65 years (Federal Statistical Office, 2022b).

The persistent link between poverty and disadvantaged health outcomes among adults and children has been well documented by national (Lampert and Kuntz, 2019; Pförtner and Schumann, 2016) and international studies (Gupta et al., 2007; Raphael, 2011). Nutrition plays a critical role in this association. For instance, the consumption of fruit and vegetables is inversely related to the risk of hypertension, coronary heart disease, and stroke (Boeing et al., 2012). Children and adults at risk of poverty tend to consume less fruit and vegetables than their wealthier counterparts (Grimm et al., 2012; Lampert and Kuntz, 2019). Thus, differences in the nutritional status may partly explain differences in health outcomes.

From a life course perspective, the nutrition during childhood is of particular importance. Nutritional preferences and eating habits established in childhood tend to be maintained in adulthood (Chong, 2022; Mikkilä et al., 2004). Moreover, nutrition in childhood has lasting effects on health in adulthood (Caballero, 2001; Kaikkonen et al., 2013). Children growing up in families at risk of poverty may therefore have a higher risk of negative health outcomes in adulthood, which in turn increases the risk of poverty as an adult (Mader et al., 2020). Despite these findings, there are only few studies illuminating the nutrition of children and adults living in households at risk of poverty in Germany.

While national surveys such as the German National Nutrition Survey II (NVS II) (Heuer et al., 2015), the German health interview and examination survey for adults (DEGS) (Mensink et al., 2017) and the German Health Interview and Examination Survey for Children and Adolescents (KiGGS) (Finger et al., 2015; Mensink et al., 2020) have revealed differences in the nutrition status between income or socioeconomical groups, they are not designed to provide a detailed picture of the nutrition status of families at risk of poverty. For instance, the DEGS questionnaires were available in Russian, Turkish, Serbo-Croatian, and English only (Gößwald et al., 2012), whereas migrants being born in Arabic countries such as Syria were among those with the highest at-risk of poverty rate (Federal Statistical Office, 2022a). Results of KiGGS may be influenced by a selection bias, as parents of respondents had, on average, a higher educational level compared to parents of nonrespondents (Hoffmann et al., 2018). Due to the written questionnaires, parents with reduced literacy may have lower chances to participate compared to parents with sufficient literacy skills (Lampert and Kuntz, 2019). In addition, none of these surveys covered poverty-related topics such as food insecurity.

Other studies have presented qualitative information about the relationship between deprived material circumstances and aspects of the nutrition such as meal preparation and food shopping (Lehmkühler, 2002; Pfeiffer et al., 2015; Yildiz, 2014). Few quantitative studies on people at risk of poverty have focused on specific population groups such as food bank users or homeless people in selected cities (Depa et al., 2015, 2018; Langnäse and Müller, 2001). However, a detailed picture of the nutritional situation of children and adults living in households at risk of poverty is still missing in Germany.

To gain a deeper understanding of the nutritional situation of persons living in families at risk of poverty including socioeconomic, behavioral, and attitudinal factors, and health characteristics, and to explore experiences, perspectives, and ideas regarding a healthy diet, the “Multidisciplinary study of the health and nutritional status of persons living in households at risk of poverty with children in Germany” (MEGA_kids) is conducted. In this article, the rationales for this study as well as its methods are described.

## Methods

### Study design

MEGA_kids is a mixed-methods cross-sectional study combining four study modules (Table 1):

The first module (BERNA, “Nutritional survey among individuals living in households at risk of poverty with children”) contains a cross-sectional survey among children, youths, and adults living in households at risk of poverty. By this module, food intake, food insecurity as well as other nutritional and health aspects such as cooking skills, nutritional knowledge, and subjective health is assessed by standardized questionnaires. In addition, participants are asked to collect their cash receipts of food shopping over a period of 2 weeks.
For the second module (IDEE, “Identification of factors influencing food security and nutritional quality from the perspective of adults living in households at risk of poverty with children”) a subsample of adults from BERNA is drawn with whom semistructured interviews are conducted about the food provision and preparation in everyday life as well as the social and health dimension of nutrition in their household.
For the third module (BEA, “Needs of households at risk of poverty with children and approaches to develop behavioral and environmental support measures for a healthy diet“), another subsample of adults from BERNA is drawn and focus groups using the World Café technique are conducted to identify opinions and experiences, and generate ideas for tailored support measures for a healthy diet from the perspective of the adult target group.
Finally, the fourth module (KENNER, “Knowledge and usage of nutrition-related preventive measures among adults of households with children at risk of poverty”) is a cross-sectional survey among parents living in households at risk of poverty about their knowledge regarding and usage of existing nutrition-related measures.

**Table 1.** Modules of MEGA_kids.

**Name**	**Sampling**	**Design**	**Duration**	**Sample size**
BERNA (“Nutritional survey among individuals living in households at risk of poverty with children”)	Multistage, partly randomized	Standardized survey (self-administered questionnaires + collecting cash receipts over 2 weeks)	ca. 20–40 min	1000 individuals (500 adults and 500 children) of 500 households
KENNER (“Knowledge and usage of nutrition-related preventive measures among adults of households at risk of poverty with children”)	Multistage, partly randomized	Standardized survey (online-questionnaire/standardized interview)	ca. 20 min	200 adults of 200 households
IDEE (“Identification of factors influencing food security and nutritional quality from the perspective of adults living in households at risk of poverty with children”)	Convenience, subsample von BERNA	Semistructured individual interview	ca. 60–120 min	20 adults of 20 households
BEA (“Needs of households at risk of poverty with children and approaches to develop behavioral and environmental support measures for a healthy diet”)	Convenience, subsample of BERNA	Participatory World Café discussions in small groups	ca. 120 min	2 × 20 adults of 2 × 20 households

### Study population

#### Quantitative module BERNA

The study population comprises children aged between 1 and 17 years as well as adults over the age of 18 years living in households at risk of poverty with children in rural and nonrural districts in Germany.

To illustrate the wider range of income-related risk of poverty, eligible households must meet at least one of the following criteria:

- household has an equivalized disposable income at or below the at risk of poverty thresholds (60% of the national median equivalized disposable income) and/or
- household head receives social transfers including, for instance, unemployment pay II, and/or
- household receives food assistance from a food bank.

Households of separated parents with shared custody are able to participate if at least one child lives for at least 15 of 28 days in this household. In households with more than one adult, the adult who is responsible for the majority of food shopping is allowed to participate.

The adult has to be sufficiently able to speak or write German, English, or Arabic. Adolescents must be able to speak or write German.

Information for children between 1 and 11 years are obtained by adults (parents), whereas adolescents between 12 and 17 years can participate themselves.

Households headed by a pregnant woman are excluded from participation. In addition, persons living in “collective living quarters” or other shared accommodations are excluded.

The sample of BERNA is expected to include 500 adults and 500 children aged between 1 and 17 years.

#### Qualitative modules BEA and IDEE and quantitative module KENNER

The qualitative modules BEA and IDEE and the quantitative module KENNER apply the same inclusion and exclusion criteria, but only adults who are sufficiently able to speak German are eligible to participate.

In BEA, one World Café is conducted with adults living in a rural district and another with adults living in a nonrural district. The sample of IDEE interview partners should include eight participants living in nonrural districts as well as six each in rural districts with a good and a worse socioeconomic condition. The sample of KENNER is expected to include 200 adult participants.

### Procedure

The data collection period of the MEGA_kids projects started in April 2022 and will be finished in June 2023.

#### Quantitative module BERNA

Participants of the BERNA survey are enrolled in three steps. In the first step, eleven districts throughout Germany are randomly drawn, stratified by the typology for rural areas of the Thünen Institute (Küpper, 2016). By combining the two dimensions rurality and socioeconomic condition, the Thünen Institute assigned each district to one of the following five types: nonrural, very rural and good socioeconomic condition, rural and good socioeconomic condition, very rural and worse socioeconomic condition and rural and worse socioeconomic condition (Küpper, 2016). For the purpose of this study, very rural and rural districts are combined resulting in three strata. Overall, five nonrural, three very rural/rural districts with good socioeconomic condition and three very rural/rural districts with worse socioeconomic condition are randomly drawn.

In the second step, facilities serving low-income population groups including, for instance, food banks (“Tafel”), job centers, family centers, social supermarkets, facilities providing employment measures for long-term unemployed individuals, and others are selected in each district. In addition, in each nonrural and very rural/rural district with worse socioeconomic condition, a random sample of five childcare facilities is drawn. Managers of all selected facilities receive written information about the study including its aim and procedure and are asked to allow the recruitment of families visiting the facility. A sampled rural district with good socioeconomic condition is included if at least one food bank and one other social facility are willing to participate and rural districts with worse socioeconomic condition and a nonrural area is included if at least one food bank, one childcare facility, and two social facilities agree to participate in the study. In doing so, a minimum of 38 facilities are included in the study.

In the third step, individuals visiting the facilities are recruited by active and/or passive recruitment strategies. In facilities with a high traffic of families being at risk of poverty such as food banks, job centers and most social supermarkets, a combination of both recruitment strategies is applied. Each facility that agreed to participate is visited twice by trained study staff. Before the first visit, facilities’ officials are asked to hang out posters and leaflets describing the study and advertising the upcoming visit of the study team. During the visit, all adults visiting the facility are informed about the aim, the study procedures, and data protection measures, verbally and by a printed study information sheet. People willing to participate are screened for inclusion into the study using a checklist. In households with more than one child, the oldest child between 1 and 17 years is able to participate. Parents willing to participate are asked to complete the informed consent form and are given the study materials including a short instruction, the study information for parents and, if applicable, for youths, all questionnaires, and a small bag for collecting cash receipts. Parents are asked whether they are available 2 weeks later to return the answered questionnaires and the collected cash receipts. If parents are not available at this date, they are given a stamped return envelope. Two weeks later, the facilities are visited again. Answered questionnaires are controlled for completeness and plausibility. People, who have not visited the facility during the first visit, are invited to participate during the second visit. If eligible and willing to participate, study materials are handed out and the participants are given a stamped return envelope.

In facilities with lower traffic of families such as counseling centers or those with unspecific traffic of families, such as kindergartens, leaflets are given to staff and they are asked to hand out the leaflet to families. The leaflets briefly describe the study procedure and provide contact details. Families willing to participate are screened by phone or by e-mail and the study materials are sent by post.

Participants receive a remuneration of 7,50 Euro for completing the parents’ version of the questionnaires, additional 7,50 Euro for completing the child/youth version of the questionnaires and additional 5 Euro for collecting cash receipts.

#### Qualitative modules BEA and IDEE

Participants of the module BERNA who have consented to be contacted regarding a qualitative module are called or contacted via e-mail depending on the details they provide. Depending on their availability via phone, call attempts can be repeated up to two times within a 2-week period or at a more convenient time as expressed by the participant. Participants are then informed about either IDEE or BEA, receive a written information about the study via e-mail, and are asked about their preferences regarding the date of the interview or World Café.

For IDEE, districts assigned to the three strata with at least three BERNA participants interested each are chosen first for interview planning. If less than three interviews can be conducted in those districts, participants from other districts assigned to the same stratum are contacted. Participants are offered the options of a telephone interview or an interview at a local venue close to their neighborhood for a one-on-one conversation. They receive a mobile text or a phone call as a reminder prior to the agreed-upon date and give written consent sent by post or at the physical meeting, respectively.

For BEA, the rural and the nonrural district with most BERNA participants interested in participating in a qualitative study are chosen as the location for the World Café event. The date and time for which most participants in a district are available is chosen at a venue close by. All participants receive a reminder via phone call or mobile text 1 week as well as 2 days prior to the event. They are informed about the conditions of the study again at the event and are asked to give their written consent before the start of the event.

After finishing the interview or the World Café, participants receive a remuneration of 30 Euro in cash or as a voucher for a supermarket of their choice.

#### Quantitative module KENNER

Similar to BERNA, participants in the KENNER survey are also enrolled in three steps. At first, one district for each of the three strata is randomly drawn. Then, food banks in these districts are purposively sampled and food bank managers receive written information about the study and are asked to allow the recruitment of adults with children in their facility. Food banks that agree to participate are visited by trained study staff. All adults visiting the facility during the visit of the study staff are verbally informed about the aim, the study procedures, and data protection measures. Individuals willing to participate are screened for inclusion into the study using a checklist.

Participants receive a remuneration of 5 Euro for completing the questionnaire.

### Data collection and data collection instruments

#### Quantitative module BERNA

The data collection method of the module BERNA consists of self-administered questionnaires for parents and for youths aged between 12 and 17 years. Data of younger children are assessed by the parents. Participants needing assistance in answering the questionnaires and participants with low literacy or other obstacles in reading/writing are interviewed.

Parents are asked to collect cash receipts of their food shopping over a period of 2 weeks.

Table 2 demonstrates the variables assessed by the questionnaires. Sociodemographics of parents and sociodemographics of children and youths as well as diverse health and nutritional aspects are assessed by a slightly adapted version of questionnaires from the KiGGS (Hölling et al., 2012) and the DEGS (Scheidt-Nave et al., 2012). Adaption was necessary to cover, for instance, all family forms (e.g. initial answer options of the question “With whom does your child mainly live?” only include heterosexual partners) and to cover school meals and meals in daycare facilities (by adding “at the day care center (e.g. KITA) or at school” into the initial question “Does your child have the option of getting a warm lunch at school?”).

**Table 2.** Variables assessed by standardized questionnaires per household in the quantitative BERNA module.

	**Unit**				
**Domains**	**Adult respondent**	**Both parents**	**Child aged 1–11 years^1^**	**Youth aged 12–17 year**	**Household**
**Sociodemographics**	Age, sex	Country of birth, year of immigration, nationality, employment status, parental occupation, educational level	Age, sex, country of birth, year of immigration, visited childcare facility/type of school attended	Age, sex, country of birth, year of immigration, visited childcare facility/type of school attended	Number of adults and children living in the household, income
**Health and health behavior**	Subjective health status, chronic diseases, physical activity	Smoking status, weight, and height	Subjective health status (parents’ perspective), weight and height, physical activity	Smoking status, subjective health status, weight and height, physical activity	
**Nutritional aspects**	Aspects of nutrition knowledge, subjective cooking skills and source of learning cooking skills, planning food shopping, planning meal preparation, frequency of cooking, social dimensions of food insecurity		Usage of childcare/school meals including frequency and reasons for nonusage, meal participation in youth clubs	Usage of school meals including frequency and reasons for nonusage, meal participation in youth clubs	Food insecurity (past 30 days), family meals including type and frequency, usage of “Tafel” food bank including frequency and duration of use, usage of soup kitchen
**Diet**	Food Frequency Questionnaire		Food Frequency Questionnaire	Food Frequency Questionnaire	

^1^ Data of children aged between 1 and 11 years are assessed by the parents.

Subjective cooking skills and source of learned cooking skills are assessed by slightly adapted questions of the computer-assisted personal interview applied in the NVS II (Max Rubner-Institut, 2005a). For instance, for convenience reasons, answer options “my mother” and “my father” were combined into “my mother / my father.” Aspects of nutrition knowledge are assessed by the questionnaires from the NVS II (Max Rubner-Institut, 2005b, 2008).

Household food insecurity over the past 30 days is measured using the Food Insecurity Experience Scale (FIES) provided by the Food and Agriculture Organization (FAO) of the United Nations (Cafiero et al., 2016). The FIES consists of eight questions and covers the access dimension of food insecurity including psychological (e.g. worrying about not having enough food; “During the past 30 days was there a time when you or others in your household worried about not having enough food to eat because of a lack of money?”), qualitative (e.g. eating only a few kinds of food), and quantitative aspects of food insecurity (e.g. skipping a meal). Answer options include “yes,” “no,” “don’t know,” and “refused.” As recommended by the FAO, statistical validation will be conducted to test whether the data are consistent with the theoretical construct the FIES is based on (Cafiero et al., 2016). Based on the number of affirmative responses, each respondent will then be assigned to categories of food insecurity. To assess the social domain of food insecurity, four questions of the Four Domain Food Insecurity Scale are used (e.g. feeling embarrassed) (Johnson et al., 2020). Answer options include “agree a lot,” “agree a little,” “disagree a little,” and “disagree a lot.” Respondents with zero affirmative answers (“agree a lot” or “agree a little”) will be categorized as social food secure, respondents with one to three affirmative answers will be categorized as mildly social food insecure and respondents who confirm all four questions will be categorized as severely social food insecure.

Planning food shopping and food preparation are assessed by three items each (e.g. making a shopping list to guide food purchases; planning cooking in advance) of the Food Related Lifestyle Inventory (Scholderer et al., 2004).

For the assessment of adults’ diet the semiquantitative food frequency questionnaire (FFQ) of the DEGS study (Scheidt-Nave et al., 2012) is used and for children and youths, the FFQ of the KiGGS is applied (Hölling et al., 2012). The development of the KiGGS FFQ is based on the DEGS FFQ, details are described elsewhere (Mensink and Burger, 2004). In short, the KiGGS FFQ “What does your child eat” covers 48 food items including beverages, the youth version “What do you eat?” and the DEGS FFQ covers 54 food items including alcoholic beverages. All versions ask the average food frequency over the past 4 weeks and the average consumed portion size per food item. Categories for frequencies are identical for all food items ranging from never to more than five times a day. Food specific portion sizes are presented by illustrations and/or by household measures such as glasses, cups, and spoons.

#### Qualitative module IDEE

A semistructured interview guide is used that includes topics related to grocery shopping and further ways of food provision, meal preparation, priorities and challenges related to nutrition in the family, financial aspects, assumptions about healthy nutrition, further roles of foods and meals (e.g. social and cultural aspects) and thoughts about how families can be supported in terms of their nutrition and related areas of life (Table 3).

**Table 3.** Themes of the IDEE interview guide.

**Main themes in IDEE**	**Complements** **(questions asked if not already elaborated by interviewee)**
(1) Everyday meals and meal preparation	Subjective priorities of interviewee and family members Ways of meal preparation Challenges in meal preparation Desired changes of diet Communal meals Eating out-of-home Child's desire to eat away from home
(2) Grocery shopping or other kinds of food provision and priorities	Challenges in food provision Criteria for decision making Strategies in case of temporarily lower budget Changes if budget would be larger
(3) Ideas about what is considered healthy nutrition	Healthy eating in the family Challenges for a healthy diet Situations when healthy eating is not priority Potential problems and solutions when wanting to increase intake of fruits and vegetables Problems and solutions when reducing sugar intake
(4) Further roles of eating in personal life	Potential functions of foods beyond nutrition Role of snacks
(5) Food and eating in social life	Occasions of eating with others, potential concerns If hosting guests: serving food, concerns or priorities Effects of financial situation on social life
(6) Measures to ease the burden for families	Means to facilitate everyday life Possible measures by the government Ranking of nutrition among current personal concerns
(7) Nutrition-related support measures	Situations where measures are needed Persons or institution who should offer support

#### Qualitative modules BEA

The World Café design (Brown et al., 2005) is used to identify experiences about healthy nutrition in everyday life from the perspective of parents and explore approaches to develop support measures. The moderator of the forum introduces participants to the World Café technique. Groups of three up to five participants sit around four tables, together with a table host. At first, all participants freely discuss about “good nutrition” (e.g. “What is good nutrition for you?”) for 7 minutes. Then, as described in Table 4, predefined questions relating to the following four themes are discussed: (1) barriers of a healthy diet, (2) facilitators of a healthy diet, (3) facilitators when adopting the perspective of a future moment, and (4) wishes concerning healthy nutrition and an elaboration of a given list of possible behavioral and environmental preventive measures. Each table host has a different set of open-ended questions relating to one of the four themes. After 20 minutes, participants change the table and mix up to a new discussion group.

**Table 4.** Discussion guide of the BEA World Café.

**Main themes in BEA**
What is good nutrition for you?
2) What makes it easier for you and your family to eat healthy in everyday life?
3) What makes it difficult sometimes for you and your family to eat well?
4) Let's assume we would have a look into the future: Today in 2 years you notice your family eats much healthier. What has changed? (Circumstances, behavior)
5) (A) If anything was possible: What would you wish for so that you and your family can eat better?
(B) Would you make use of the following support options? If yes: What would be important? If no: why rather not? Free of charge cooking class or workshop in your neighborhood Free of charge breakfast and lunch in schools and daycare centers Free of charge lunch for children during school holidays Free of charge or affordable warm meals, e.g. at a food bank More affordable fruits and vegetables for low-income families Nutritional counseling and presentations about nutrition

#### Quantitative module KENNER

An online survey (EFS Survey, Tivian XI GmbH) is performed on-site by using tablet computers. Participants needing assistance in using a tablet computer and participants with low literacy are interviewed.

Sociodemographics and health aspects are assessed by a slightly adapted version of questionnaires from the DEGS (Scheidt-Nave et al., 2012). Attitudes toward healthy nutrition (e.g. “Eating healthily is an important determinant of a long and healthy life” and “I am tired of listening what I should eat or should not eat”) are assessed by eight simplified items of Diehl's scale “Attitudes towards healthy nutrition” using a five-point Likert scale (strongly disagree to strongly agree) (Diehl, 2006). Questions regarding knowledge and usage of different types of measures were developed with various answer options such as recipe apps, nutrition-related websites, and school or childcare facility nutrition projects. In addition, specific programs or measures to promote a healthy diet are shown with the corresponding logo, for example, existing mobile applications, or governmental websites promoting activities around a healthy diet. Depending on the responses, participants are asked about reasons of nonusage or about evaluative aspects of the specific measures including, for instance, access and layout, comprehensibility, and suitability. Similarly, the awareness and practice of the dietary recommendations of the German Nutrition Society (DGE) are surveyed (“5 a day,” “10 guidelines for a wholesome diet,” “DGE nutrition circle,” and “The German Three-Dimensional Food Pyramid”).

Finally, participants are asked about topics they wish preventive measures to be related, their preferred type of nutrition-related preventive measures and the preferred way to be informed about such offers.

### Analyzes

#### Quantitative module BERNA

Descriptive analyzes provide information on the distribution of the nutritional and health aspects including nutrition-related expenses and the diet of individuals living in families at risk of poverty according to sociodemographic characteristics such as age, sex, origin/migration background, parental educational level, parental occupation and living in rural and urban areas. Mean, standard deviation, and median will be provided for continuous variables and absolute and relative frequencies with 95% confidence intervals based on bootstrapping will be given for categorical variables. Absolute amount of food consumption will be provided in gram per day or gram per week. In addition, the proportion of participants achieving cut-offs of the age- and sex-specific recommendations (for children and youths: “Optimised mixed diet” (Kersting et al., 2005), for adults: recommendations of the German Nutrition Society (Oberritter et al., 2013)) will be calculated. The Healthy Nutrition Score HuSKY will be calculated for adolescents (Kleiser et al., 2009). Relationships between the diet, food insecurity and diverse nutritional and health aspects will be analyzed by multivariate analyzes taking the clustering of the data into account (generalized linear mixed models and linear mixed models).

#### Qualitative modules BEA and IDEE

Interviews and World Café discussions are digitally recorded, transcribed verbatim, and pseudonymized. Qualitative content analysis will be applied, following both a deductive and an inductive approach (Mayring, 2019). Data from the two qualitative modules are analyzed separately. MAXQDA software will be used for the analysis.

#### Quantitative module KENNER

The mean, standard deviation, minimum, and maximum are given for continuous variables. Absolute and relative frequencies are provided for categorical variables. Associations between variables are investigated by corresponding association measures (chi-square tests including Phi and Cramer's V for categorical variables, eta-coefficient for a nominal and a continuous variable, Spearman correlation coefficient for ordinal variables and Pearson for continuous variables).

## Discussion

For the first time, MEGA_kids will provide a more comprehensive picture of the dietary behavior, food insecurity, nutritional expenses, subjective facilitators and barriers of a healthy diet, and diverse nutritional and health aspects among individuals living in families at risk of poverty in Germany.

A major strength of MEGA_kids is the application of an innovative mixed-methods approach combining quantitative and qualitative research methods and allowing wide-ranging and deep investigations of nutritional themes among the target group. The application of a thoroughly planned recruitment strategy involving proactive and passive elements and diverse specific recruitment measures (e.g. multilingual study team, culturally sensitive recruitment materials) enables the participation of people who are usually underrepresented in national surveys and are considered hard to reach (Shaghaghi et al., 2011). Several measures are applied to reduce the risk of selection bias such as randomization, the combination of proactive and passive recruitment strategies, the inclusion of a wide range of facilities, and regular trainings of the recruitment staff.

However, some limitations of the mixed-methods study design have to be considered. First, the cross-sectional design of the modules will not allow the determination of cause and effect. Second, due to the facility-based recruiting strategy the sample is limited to families using any of the facilities; little information is available of families that are at risk of poverty but do not use any of the facilities included. To minimize the risk of selection bias, a broad range of facilities is included. Third, parents participating in the qualitative module must be able to speak German. However, individuals with migration background have a particular high risk of being at risk of poverty (Federal Statistical Office, 2023) and families with insufficient skills in the languages provided may be underrepresented in the qualitative modules of MEGA_kids. In addition, the readiness or refusal to participate in an interview or group discussions might reflect differences within this group that cannot be explored within the qualitative modules and might introduce some element of selection bias.

Despite these limitations, MEGA_kids will considerably enlarge the knowledge related to the nutritional situation of families with children at risk of poverty.

MEGA_kids will

- provide data for epidemiological research; data of the quantitative module BERNA will be made publicly available;
- guide officials to target and prioritize public health measures;
- inform policy makers to implement and improve healthy policies;
- identify research gaps to be prioritized; and
- finally, help to improve the nutrition and health of families living at risk of poverty.

First results of MEGA_kids will be published from the end of 2024 in the Nutrition Report of the German Society of Nutrition. Detailed results will then be published in national and international scientific journals.

## References

[bibr1-02601060241242159] Boeing H Bechthold A Bub A , et al. (2012) Critical review: Vegetables and fruit in the prevention of chronic diseases. European Journal of Nutrition 51(6): 637–663. 22684631 10.1007/s00394-012-0380-y PMC3419346

[bibr2-02601060241242159] Caballero B (2001) Early nutrition and risk of disease in the adult. Public Health Nutrition 4(6): 1335–1336. 11918474 10.1079/phn2001212

[bibr3-02601060241242159] Cafiero C Nord M Viviani S , et al. (2016) Voices of the Hungry. Methods for Estimating Comparable Prevalence Rates of Food Insecurity Experienced by Adults throughout the World. Rome: FAO.

[bibr4-02601060241242159] Chong MF-F (2022) Dietary trajectories through the life course: Opportunities and challenges. The British Journal of Nutrition 128(1): 154–159. 35475441 10.1017/S0007114522001295

[bibr5-02601060241242159] Depa J Gyngell F Müller A , et al. (2018) Prevalence of food insecurity among food bank users in Germany and its association with population characteristics. Preventive Medicine Reports 9: 96–101. 29527460 10.1016/j.pmedr.2018.01.005 PMC5840845

[bibr6-02601060241242159] Depa J Hilzendegen C Tinnemann P , et al. (2015) An explorative cross-sectional study examining self-reported health and nutritional status of disadvantaged people using food banks in Germany. International Journal for Equity in Health 14: 141, 1–10. 10.1186/s12939-015-0276-6 PMC4658762 26601718

[bibr7-02601060241242159] Diehl JM (2006) Fragebögen zur Erfassung ernährungs- und gewichtsbezogener Einstellungen und Verhaltensweisen. Giessen: Department of Psychology University of Giessen.

[bibr8-02601060241242159] Federal Statistical Office (2022a) Bevölkerung und Erwerbstätigkeit. Bevölkerung mit Migrationshintergrund - Ergebnisse des Mikrozensus 2021. Fachserie 1 Reihe 2.2. Wiesbaden: Federal Statistical Office.

[bibr9-02601060241242159] Federal Statistical Office (2022b) Living conditions, risk of poverty. Available at: https://www.destatis.de/EN/Themes/Society-Environment/Income-Consumption-Living-Conditions/Living-Conditions-Risk-Poverty/_node.html (accessed 13 February 2023).

[bibr10-02601060241242159] Federal Statistical Office (2023) Migration and integration. Available at: https://www.destatis.de/EN/Themes/Society-Environment/Population/Migration-Integration/_node.html (accessed 24 March 2023).

[bibr11-02601060241242159] Finger JD Varnaccia G Tylleskär T , et al. (2015) Dietary behaviour and parental socioeconomic position among adolescents: The German health interview and examination survey for children and adolescents 2003–2006 (KiGGS). BMC Public Health 15: 498. 25985772 10.1186/s12889-015-1830-2 PMC4492169

[bibr12-02601060241242159] Gößwald A Lange M Kamtsiuris P , et al. (2012) [DEGS: German health interview and examination survey for adults. A nationwide cross-sectional and longitudinal study within the framework of health monitoring conducted by the Robert Koch Institute]. Bundesgesundheitsblatt, Gesundheitsforschung, Gesundheitsschutz 55(6–7): 775–780. 22736155 10.1007/s00103-012-1498-z

[bibr13-02601060241242159] Grimm KA Foltz JL Blanck HM , et al. (2012) Household income disparities in fruit and vegetable consumption by state and territory: Results of the 2009 behavioral risk factor surveillance system. Journal of the Academy of Nutrition and Dietetics 112(12): 2014–2021. 23174688 10.1016/j.jand.2012.08.030

[bibr14-02601060241242159] Gupta RP-S de Wit ML McKeown D (2007) The impact of poverty on the current and future health status of children. Paediatrics & Child Health 12(8): 667–672. 19030444 10.1093/pch/12.8.667 PMC2528796

[bibr15-02601060241242159] Heuer T Krems C Moon K , et al. (2015) Food consumption of adults in Germany: Results of the German National Nutrition Survey II based on diet history interviews. The British Journal of Nutrition 113(10): 1603–1614. 25866161 10.1017/S0007114515000744 PMC4462161

[bibr16-02601060241242159] Hoffmann R Lange M Butschalowsky H , et al. (2018) KiGGS Wave 2 cross-sectional study—participant acquisition, response rates and representativeness. Journal of Health Monitoring 3(1): 78–91. 35586176 10.17886/RKI-GBE-2018-032 PMC8848911

[bibr17-02601060241242159] Hölling H Schlack R Kamtsiuris P , et al. (2012) [The KiGGS study. Nationwide representative longitudinal and cross-sectional study on the health of children and adolescents within the framework of health monitoring at the Robert Koch Institute]. Bundesgesundheitsblatt, Gesundheitsforschung, Gesundheitsschutz 55(6–7): 836–842. 22736165 10.1007/s00103-012-1486-3

[bibr18-02601060241242159] Johnson CM Ammerman AS Adair LS , et al. (2020) The Four Domain Food Insecurity Scale (4D-FIS): Development and evaluation of a complementary food insecurity measure. Translational Behavioral Medicine 10(6): 1255–1265. 33421083 10.1093/tbm/ibaa125 PMC7796713

[bibr19-02601060241242159] Kaikkonen JE Mikkilä V Magnussen CG , et al. (2013) Does childhood nutrition influence adult cardiovascular disease risk?—insights from the young Finns study. Annals of Medicine 45(2): 120–128. Taylor & Francis: 120–128. 22494087 10.3109/07853890.2012.671537

[bibr20-02601060241242159] Kersting M Alexy U Clausen K (2005) Using the concept of food based dietary guidelines to develop an optimized mixed diet (OMD) for German children and adolescents. Journal of Pediatric Gastroenterology and Nutrition 40(3): 301–308. 15735483 10.1097/01.mpg.0000153887.19429.70

[bibr21-02601060241242159] Kleiser C Mensink G Scheidt-Nave C , et al. (2009) HuSKY: A healthy nutrition score based on food intake of children and adolescents in Germany. The British journal of nutrition 102(4): 610–618. 10.1017/S0007114509222689 19203423

[bibr22-02601060241242159] Küpper P (2016) *Abgrenzung und Typisierung ländlicher Räume*. Thünen Working Paper 68. Braunschweig: Thünen-Institut. Available at: https://literatur.thuenen.de/digbib_extern/dn057783.pdf (accessed 20 April 2018).

[bibr23-02601060241242159] Lampert T Kuntz B (2019) [Effects of poverty for health and health behavior of children and adolescents : Results from KiGGS wave 2]. Bundesgesundheitsblatt, Gesundheitsforschung, Gesundheitsschutz 62(10): 1263–1274. 31529186 10.1007/s00103-019-03009-6

[bibr24-02601060241242159] Langnäse K Müller MJ (2001) Nutrition and health in an adult urban homeless population in Germany. Public Health Nutrition 4(3): 805–811. Cambridge University Press: 805–811. 11415488 10.1079/phn2000119

[bibr25-02601060241242159] Lehmkühler S (2002) Die Gießener Ernährungsstudie über das Ernährungsverhalten von Armutshaushalten (GESA) - qualitative Fallstudien. Gießen: University of Gießen.

[bibr26-02601060241242159] Mader S Rubach M Schaecke W , et al. (2020) Healthy nutrition in Germany: A survey analysis of social causes, obesity and socioeconomic status. Public Health Nutrition 23(12): 2109–2123. 32338236 10.1017/S1368980019004877 PMC10200647

[bibr27-02601060241242159] Max Rubner-Institut (2005a) CAPI-Interview der Nationalen Verzehrsstudie (NVS) II. Available at: https://www.mri.bund.de/fileadmin/MRI/Institute/EV/NVS_II_CAPI_Interview.pdf (accessed 1 October 2021).

[bibr28-02601060241242159] Max Rubner-Institut (2005b) Fragebogen der Nationalen Verzehrsstudie (NVS) II. Available at: https://www.mri.bund.de/fileadmin/MRI/Institute/EV/NVS_II_Fragebogen.pdf (accessed 1 October 2021).

[bibr29-02601060241242159] Max Rubner-Institut (2008) Nationale Verzehrsstudie II, Ergebnisbericht, Teil 1. Die bundesweite Befragung zur Ernährung von Jugendlichen und Erwachsenen. Karlsruhe: Max Rubner-Institut.

[bibr30-02601060241242159] Mayring P (2019) Qualitative Inhaltsanalyse. In: Mey G Mruck K (eds) Handbuch Qualitative Forschung in der Psychologie. Springer Reference Psychologie. Wiesbaden: Springer Fachmedien, 1–17. Available at: 10.1007/978-3-658-18387-5_52-2 (accessed 24 April 2023).

[bibr31-02601060241242159] Mensink GBM Burger M (2004) [What do you eat?] Bundesgesundheitsblatt - Gesundheitsforschung -Gesundheitsschutz 47(3): 219–226. 15205789 10.1007/s00103-003-0794-z

[bibr32-02601060241242159] Mensink GBM Haftenberger M lage Barbosa C , et al. (2020) EsKiMo II - Die Ernährungsstudie als KiGGS-Modul. Berlin: Robert Koch-Institut.

[bibr33-02601060241242159] Mensink GBM Schienkiewitz A Lange C (2017) Gemüsekonsum bei Erwachsenen in Deutschland. Journal of Health Monitoring 2(2), RKI-Bib1 (Robert Koch-Institut).

[bibr34-02601060241242159] Mikkilä V Räsänen L Raitakari OT , et al. (2004) Longitudinal changes in diet from childhood into adulthood with respect to risk of cardiovascular diseases: The cardiovascular risk in young Finns study. European Journal of Clinical Nutrition 58(7): 7. Nature Publishing Group: 1038–1045. 10.1038/sj.ejcn.1601929 15220946

[bibr35-02601060241242159] Oberritter H Schäbethal K Ruesten A , et al. (2013) The DGE nutrition circle—presentation and basis of the food-related recommendations from the German Nutrition Society (DGE). Ernahrungs Umschau 60: 24–29.

[bibr36-02601060241242159] Pfeiffer S Ritter T Oestreicher E (2015) Food insecurity in German households: Qualitative and quantitative data on coping, poverty consumerism and alimentary participation. Social Policy and Society 14(03): 1–13.

[bibr37-02601060241242159] Pförtner T-K Schumann N (2016) [Poverty, public transfers and health: An analysis on self-rated health of social benefit recipients in Germany]. Gesundheitswesen (Bundesverband Der Arzte Des Offentlichen Gesundheitsdienstes (Germany)) 78(8–09): e69–e79. 10.1055/s-0035-1564206 26630447

[bibr38-02601060241242159] Raphael D (2011) Poverty in childhood and adverse health outcomes in adulthood. Maturitas 69(1): 22–26. 21398059 10.1016/j.maturitas.2011.02.011

[bibr39-02601060241242159] Scheidt-Nave C Kamtsiuris P Gößwald A , et al. (2012) German Health interview and examination survey for adults (DEGS)—design, objectives and implementation of the first data collection wave. BMC Public Health 12: 730. 22938722 10.1186/1471-2458-12-730 PMC3490742

[bibr40-02601060241242159] Scholderer J Brunsø K Bredahl L , et al. (2004) Cross-cultural validity of the food-related lifestyles instrument (FRL) within Western Europe. Appetite 42(2): 197–211. 15010184 10.1016/j.appet.2003.11.005

[bibr41-02601060241242159] Shaghaghi A Bhopal RS Sheikh A (2011) Approaches to recruiting ‘hard-to-reach’ populations into Re­search: A review of the literature. Health Promotion Perspectives 1(2): 86–94. 24688904 10.5681/hpp.2011.009 PMC3963617

[bibr42-02601060241242159] Brown J Isaacs D and World Café Community. (2005) The World Café: Shaping Our Futures Through Conversations That Matter. San Francisco, CA: Berrett-Koehler Publishers.

[bibr43-02601060241242159] Yildiz J (2014) Auswirkungen eines verringerten Haushaltsbudgets auf den Lebensmittelkonsum. Eine empirische Untersuchung zu den finanziell bedingten Veränderungen am Lebensmitteleinkauf und beim Ernährungsverhalten. Gießen: Justus-Liebig-Universität Gießen.

